# Procedures for Fecal Microbiota Transplantation in Murine Microbiome Studies

**DOI:** 10.3389/fcimb.2021.711055

**Published:** 2021-09-21

**Authors:** Suresh C. Bokoliya, Yair Dorsett, Hunter Panier, Yanjiao Zhou

**Affiliations:** Department of Medicine, University of Connecticut (UConn) Health, Farmington, CT, United States

**Keywords:** fecal microbiota transplantation, mice, microbiome, engraftment, procedure

## Abstract

Fecal microbiota transplantation (FMT) has been widely recognized as an approach to determine the microbiome’s causal role in gut dysbiosis-related disease models and as a novel disease-modifying therapy. Despite potential beneficial FMT results in various disease models, there is a variation and complexity in procedural agreement among research groups for performing FMT. The viability of the microbiome in feces and its successful transfer depends on various aspects of donors, recipients, and lab settings. This review focuses on the technical practices of FMT in animal studies. We first document crucial factors required for collecting, handling, and processing donor fecal microbiota for FMT. Then, we detail the description of gut microbiota depletion methods, FMT dosages, and routes of FMT administrations in recipients. In the end, we describe assessments of success rates of FMT with sustainability. It is critical to work under the anaerobic condition to preserve as much of the viability of bacteria. Utilization of germ- free mice or depletion of recipient gut microbiota by antibiotics or polyethylene glycol are two common recipient preparation approaches to achieve better engraftment. Oral-gastric gavage preferred by most researchers for fast and effective administration of FMT in mice. Overall, this review highlights various methods that may lead to developing the standard and reproducible protocol for FMT.

## 1 Introduction

The human microbiota harbors an extensive reservoir of genetic information encoded within ~10 to 100 trillion microbial cells present throughout the human body. Most of this genetic information is present within the human gastrointestinal tract and is commonly referred to as our “second genome”. The ~ 1100 gut microbial species are central to the proper development and function of numerous physiological processes such as sustaining homeostasis in gut barrier integrity (Shi et al., 2017), nutrition metabolism (Nicholson et al., 2012), host immunity (Olszak et al., 2012) and the regulation of neuropsychological behaviors (Diaz Heijtz et al., 2011).

Microbiome research has made tremendous strides in the past decade. After the initial characterization of the microbiome in the healthy population from the Human Microbiome Project (Human Microbiome Project, 2012a; Human Microbiome Project, 2012b), accumulating data from animal and human studies have advocated that gut microbiome dysbiosis (alteration) is associated with onset, development, and progression of gastrointestinal (Chang and Lin, 2016), metabolic (Dabke et al., 2019), autoimmune (Xu et al., 2019), neurologic (Felice and O’Mahony, 2017), and psychiatric diseases (Cenit et al., 2017). Research is rapidly moving from association to causation by experimentally manipulating the gut microbiome to precisely understand the contribution and underlying mechanisms of microbiome in disease. Fecal microbiota transplantation (FMT) is one of the most commonly used approaches for investigating the causal connection between the gut microbiome and diseases in animal models. FMT has shown that the transplanted microbiome can influence physiological functions and alleviate a wide range of diseases that include *Clostridium Difficile* infection (CDI), inflammatory bowel diseases (IBD), irritable bowel syndrome (IBS), obesity, diabetes, aging, cancer, autism, multiple sclerosis (Evrensel and Ceylan, 2016), Parkinson’s disease (Sun et al., 2018), anorexia nervosa, food allergies and neurological disorders (Bajaj et al., 2017; Kang et al., 2017; Wang et al., 2018a) in animal models. These findings have spearheaded the advancement of targeted microbiome therapeutic using FMT for the treatment of human diseases.

**Figure 1:**
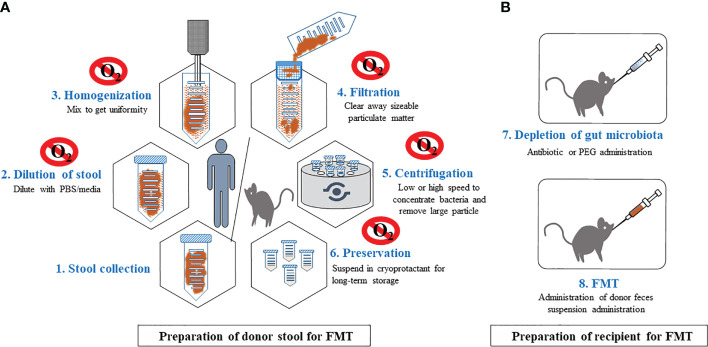
General procedures for FMT in murine. Step-wised procedures of FMT from human or mice donors to mice recipients. This includes preparation of donor stool **(A)** and preparation of recipients **(B)**. Preparation of donor stool is recommended in anaerobic environment. Raw stools will be diluted, homogenized, filtered, followed by centrifuge to remove large particles. Processed stool can be stored in long term in -800C. Recipient mice will treated with antibiotics or PEG to deplete the majority of the gut microbiota, providing an ecological niche for colonization of transplanted stool microbiota.

Although FMTs are widely used for studying causality in disease models, standard protocols for relevant FMT procedures do not exist for even basic procedures such as donor stool preparation (selection, storage and processing), delivery mode, dosage, duration of administration and recipient preparation. Such experimental variation complicates the interpretation of results across studies. In this review, we provide a detailed summary of the techniques currently utilized for FMTs in animal studies including FMT from mouse to mouse and human to mouse) (**Figure 1**) and call for standardizing protocols to increase rigor and reproducibility of critical approach for microbiome research and therapeutics.

## 2 Evidence of FMT in Modifying Disease Development

Several animal studies have demonstrated the benefits of FMT in preventing and treating diseases. Lawley’s research group reported that FMT from a healthy donor mouse into recipient mouse with CDI led to resolution of illness and infection (Lawley et al., 2012). A study by Palma et al. reported altered gastrointestinal function, activation of innate immunity, and anxiety-like behavior in germ-free (GF) mice treated with feces matter of diarrheal IBS patients (De Palma et al., 2017). FMT improves colitis symptoms in the mouse model by upregulating aryl hydrocarbon receptors (Wei et al., 2018). Ferrere and his colleagues reported the prevention of alcoholic liver disease in mice when treated with the fecal transplant of alcohol-fed donor mice resistant to alcoholic liver disease (Ferrere et al., 2017). A recent study reported decrement or limitation in the accumulation of p-cresyl sulfate and uremic toxins after FMT from healthy mice into mice with chronic kidney disease (Barba et al., 2020). FMT also reverses the course of lethal sepsis by improving pathogen clearance through the restoration of host immunity in an interferon regulatory factor 3-dependent method (Kim et al., 2020). FMT of resveratrol-fed mice enhanced glucose homeostasis and cardiovascular symptoms and reduced the high blood pressure in a mouse model of hypertension (Kim et al., 2018b). FMT also alleviates high-fat diet-induced steatohepatitis in mice *via* restoring the beneficial regulation of gut microbiota (Zhou et al., 2017). FMT might lessen obesity and digestion in both humans and mice (Ridaura et al., 2013). Bárcena’s research group found that FMT from wild-type mice improved health span and life expectancy in progeroid mice. The re-establishment of secondary bile acids is a potential mechanism for restoring a healthy microbiome (Barcena et al., 2019). A number of reports support a protecting role of specific FMT against development and progression of melanoma (Le Bastard et al., 2018; Wang et al., 2019) possibly enhancing anti-tumor immune responses mediated by increased dendritic cells activity, and improved priming of CD8 T cells in and around the tumor microenvironment.

FMT also shows encouraging results in neuropsychological diseases. FMT from resilient rats significantly reduced the pain and depression in antibiotic administered pseudo-germ-free mice (Yang et al., 2019). A recent report advocated FMT of youth gut microbiome in aged mice to reverse the weak stroke recovery (Lee et al., 2020). Remarkably, FMT inhibited the TLR4/TBK1/NF-κB/TNF-a signaling pathway, reduced gut microbial dysbiosis, decreased the activation of brain microglia and astrocytes, and alleviated physical impairment in Parkinson’s disease mice (Sun et al., 2018; Sun et al., 2019). Multiple sclerosis derived gut microbiome contains factors that precipitate numerous sclerosis in a humanized mouse model (Berer et al., 2017). FMT also contains unidentified microbes can be potentially pathogenic (Elinav et al., 2011; Food and Drug Administration, 2020) that exacerbate illness or even be fatal for recipients. Further, FMT might induce obesity as metabolic syndrome in recipients as a long-term effect (Ridaura et al., 2013). FMT can also be the risk of infection or overt immune reaction in immunocompromised recipients (Carlson, 2020). These reports suggest that FMT also has some limitations that need to be evaluated for its continued development.

## 3 Donor Stool Preparation for FMT

### 3.1 Timing of Stool Collection

Collection timing of stool is imperative given reports signifying the circadian rhythms of the abundance of gut microbiota. Diurnal rhythmicity reported in 20-83% of mice gut microbiota (Zarrinpar et al., 2014). Around 20% of human gut microbiota displayed diurnal fluctuations in average abundance (Thaiss et al., 2014). Clostridiales, Lactobacillales, and Bacteroidales, which comprise 60% of total gut microbiota, displayed acrophase and bathyphase in abundance throughout the 24-hour cycle. Noticeably, the bacterial rhythmicity was independent of cage or housing conditions (Thaiss et al., 2014).

Liang and his research group found that bacterial load was highest during night time (11:00 PM), and the lowest load was reported early morning (7:00 AM). Bacterial load steadily increased from the early light phase and decreased toward the late-night dark stage (midnight). The average higher richness (60-66%) of Bacteroidetes was noted at 11:00 PM and 11:00 AM and lower at other time points. The average higher richness (45%) of Firmicutes was noted at 3:00 AM and 7:00 AM, and the lowest richness (29%) was recorded at 11:00 PM. They also reported male mice had higher bacterial load than females, though females had higher bacterial rhythms (Liang et al., 2015). Some reports endorsed to collect mice fecal samples in the early morning between 7:00 AM to 11:00 AM (Ericsson et al., 2018) or afternoon between 3:00 PM to 5:00 PM to reduce potential circadian rhythm effects (Wang et al., 2018b). Due to mice nocturnal feeding actions, it will take less time to excrete the mice and collect the feces in the morning. For human donors, individuals delivered fresh stool one month after health screening (Bakken et al., 2011), however no specific rationale for stool collection timing was provided.

### 3.2 Frozen-Thaw Stool *vs*. Fresh Stool

Ericsson and his colleagues conducted an FMT study in mice with frozen and thawed stool in contrast with fresh stool to ensure microbes’ viability after freezing. They stated that frozen feces and fresh cecal contents both performed comparably to fresh feces as the model source for FMT (Ericsson et al., 2017). Using frozen stool permit researchers to freeze and bank multiple samples for later use. However, the number of freeze-and-thaw cycles has been described to influence microbial populations (Sergeant et al., 2012). It will be a standard lab practice to make aliquots of feces to prevent multiple freezes and thaw cycles (Thomas et al., 2015).

### 3.3 Storage Condition of Stool

Fecal samples kept at room temperature for up to twenty-four hours hold a microbiota comparable in composition to a fresh sample. Storage of feces at room temperature over 24 hours increases the abundance of Actinobacteria and decreases the abundance of Firmicutes (Choo et al., 2015). Feces sample cooled at 4°C did not vary significantly in the microbial community of the control feces samples stored at −80°C for over twenty-four hours period (Tedjo et al., 2015). However, metabolic disease feces or IBS fecal samples are less stable throughout storage at room temperature (Roka et al., 2007; Tana et al., 2010). It is tempting to speculate that higher levels of proteases and acids may result in the rapid degradation of fecal DNA. Short‐term storage of fecal material at −20°C for one month exhibited similar enteric colonization ability frozen at −80°C (Lauber et al., 2010). Long‐span storage of transplanted fecal material at −20°C for more than one month can result in uncertainty of the clinical outcome following FMT (Takahashi et al., 2019). During long-term storage at -80°C, fecal materials kept at up to six months retain a microbiota comparable in composition to a fresh sample (Carroll et al., 2012). There were reports showing that feces can maintain a steady microbial population for up to 2 years when stored at -80°C (Shaw et al., 2016). Freezing of feces at −20°C for around two months has been shown to increase the ratio of Firmicutes to Bacteroidetes (Bahl et al., 2012). Collectively, storing of stool samples streamlines the practical aspects of FMT with effectiveness or safety.

### 3.4 Preservation of Stool

Conservation of fecal transplants is essential to give rapid access to fecal material whose safety has been tested upstream in an allogenic context. Cryoprotection before freezing is a crucial step to preserve bacterial cell viability and integrity (Fadda, 2020). The currently recommended protocol advocates 10% glycerol for the conservation of frozen fecal samples (Cammarota et al., 2014). Glycerol is selected due to its low toxicity to bacterial cells intended for FMT (Hamilton et al., 2012). Storage of stool samples in normal saline causes a decline in all cultured microbiota compared to 10% glycerol solution except coliforms and *Lactobacilli*. A 10% glycerol is not appropriate for downstream lyophilization as it leads to a dry and sticky product. Cryoprotectants such as maltodextrin and trehalose can be good alternatives of glycerol+ dimethyl sulfoxide as they increase medium viscosity and limit ice crystallization and osmotic disparity during freezing. Cocktail recipes in the ratios of 3:1 maltodextrin and 1:3 trehalose in NaCl retained > 60% microbial viability in stored stool over a three-month period, a proportion which is little less to freshly prepared stool (Béal and Fonseca, 2015; Burz et al., 2019). It advised to include feces preservation method to prevent drastic change in viability when stored.

### 3.5 Stool Processing (Anaerobic *vs*. Aerobic)

The human gut consists mostly of anaerobic bacteria and sensitive to oxygen exposure. Anaerobic bacteria outnumber aerobic bacteria by a factor of 1000:1 (Finegold, 1995). Improper handling of the stool may lose the variability of many anaerobic bacteria, thus affects the effect of FMT. Approximately 61.69% of cells were alive when samples were exposed to oxygen <2 min, and cell viability decreased to 55.52% after exposure to oxygen for 90 minutes (Bellali et al., 2019). Under exposure to oxygen for < 2 minutes viability was reduced to 49% (Ben-Amor et al., 2005). Another report stated anaerobic microbes viability ~50% when the human stool was exposed to oxygen for 4-5 minutes, falling to 0.1% post 40 minutes, and no cell was stayed alive after 2 hours (Brusa et al., 1989). Papanicolas and his colleagues found around 50% bacterial viability under stringent anaerobic conditions (Papanicolas et al., 2019). Homogenization in ambient air or freeze-thaw sequence reduced microbial viability to 19% and 23%, correspondingly. Processing of fecal samples in ambient air can be a cause of multi-fold declines in the abundance of *Faecalibacterium prausnitzii* (12 fold decline), *Subdoligranulum variable* (8 fold decline), *Eubacterium hallii* (5 fold decline), and ~3 fold decline in *Ruminococcus, Roseburia, lachnospira*, and *Dorea* abundance (Papanicolas et al., 2019). Processing of fecal slurries in ambient air significantly reduced the production of short-chain fatty acids such as butyrate and acetate and the capacity for biosynthesis of important anti-inflammatory metabolites (Papanicolas et al., 2019). However, when the stool sample preserved in maltodextrin and trehalose was rapidly processed with oxygen did not affect the bacterial viability, especially for oxygen-sensitive Firmicutes phylum (Burz et al., 2019). Stool preserved in medium (patent no. N°1H53316 CAS 25 FR) containing antioxidants showed 58.51% cell viability when exposed to oxygen for 90 minutes (Bellali et al., 2019). The understanding of this notion is vital for the improvement of techniques for maintaining the viability of bacteria.

## 4 Processing of Donor Stools for FMT Preparation

### 4.1 Mouse Donor Stool Processing

There is frequently some discrepancy in FMT preparation across institutes and labs. However, the overall process is similar and includes mixing feces with a bacteriostatic fluid, eliminating particulate substance, and transporting feces to the recipient. Depending on the dosage and number of FMT, a large quantity of stool can be used for FMT preparation (Thaiss et al., 2014; Barcena et al., 2019; Stebegg et al., 2019; Sun et al., 2019). On the day of FMT, either fresh feces can be diluted in the sterilized phosphate-buffered saline (PBS) to get an estimated fecal suspension (Sivan et al., 2015; Sun et al., 2019). Frozen aliquot fecal suspension can be defrosted for around 10-15 minutes in a water bath at 37°C and appended with L-cysteine amino acid. L-Cysteine is used as a medium, reducing agent to preserve anaerobes. Mixing of feces can also be done with filtered autoclaved water (Ericsson et al., 2017), 200 proof ethanol (Lin et al., 2019) and NaCl as alternatives for PBS. It is recommended FMT should be executed within 6 hours after defrosting. Besides, it is recommended that the dilution ratio of the feces could be adjusted in FMT preparation (Rasmussen et al., 2019). The feces can be diluted approximately 3 times (Stebegg et al., 2019) to 6 times (Barcena et al., 2019) with PBS. As more than half of the stool bacteria are non-cultivable, it is hard to know the number of live bacteria in the donor samples. Vigorously shaking or homogenization of the feces suspension is endorsed to confirm proper mixing (Ericsson et al., 2017). The fecal suspension should be filtered with a filter or gauze to clear away sizeable particulate matter. Further, centrifugation can be performed to pelleting out undissolved solids matter (Liu et al., 2020). Centrifugation speed varies for FMT preparation such as 500 × *g* for 5 min, (Stebegg et al., 2019) 800 × g for 3 min, (Lai et al., 2018) 2000 ×g for 5 min (Liu et al., 2020) and 5000 × g for 20 min at 20°C. (Zhou et al., 2019) Low-speed centrifugation was probably performed to remove particulate matter, and high-speed centrifugation to concentrate bacteria.

### 4.2 Human Donor Stool Processing

Although trivial differences depend on the specific condition, most organizations prepare the stool centered on a similar protocol. Varieties of stool diluents, such as sterile NaCl, PBS, and skim milk + BHI media (Gough et al., 2011; Brandt and Aroniadis, 2013; Wrzosek et al., 2018) are used to mix feces. The fecal materials’ dilution ratio could be adjusted, considering heterogeneity in the fecal microbes between different individuals or donors (Smits et al., 2013) and advised to be diluted 3–5 times with PBS (Cammarota et al., 2017). Fresh feces should be transported on an icebox to a particular laboratory within 2 hours after excretion (Lee et al., 2016). Feces should be filtered three times through gauze (Mattila et al., 2012), cell strainer, or 0.25 mm stainless steel mesh to remove the undigested and small solid particles in the fecal suspension (Owens et al., 2013). The fecal suspension could be centrifuged 6000 × g for 15 min to remove insolubilized material (Hamilton et al., 2012; Staley et al., 2017).

Notably, all apparatus and solutions used in the fecal suspension preparation should be strictly sterilized. All fecal material preparation processes should be carried out at a room temperature of 20–30°C, preferably in an anaerobic chamber (Rossen et al., 2015).

## 5 Dosage Strategy for FMT

The best FMT dose up strategy depends on the disease and the type of bacteria to be studied. Staley and his colleagues quantified the donor’s microbial load for FMT preparations using a Petroff-Hauser counting chamber under a microscope (Staley et al., 2017). However, the attainment of FMT mostly depends on the recipient bacterial richness rather than the composition of the donor’s fecal samples. Low microbial load in the recipient can help in improved colonizing of donor microbiota (Sarrabayrouse et al., 2020). The dose-volume and duration of fecal microbiota administration range from a single dose to twice a week for numerous weeks (Hintze et al., 2014; Wrzosek et al., 2018). The scheme using only one dose of FMT was not recommended as there is a cage-dependent shift of the gut microbiota over time, potentially due to the low stability of the transplanted microbiome (Wrzosek et al., 2018). In comparison, FMT twice a week for several weeks can affect the engraftment and trouble the strength of the newly formed microbial ecosystem. FMT twice during the first week allows engraftment of sub-dominant microbes, such as *Bifidobacterium* (Wrzosek et al., 2018). FMT of the human stool to mice once a week for four weeks may be the best settlement, as it allowed the engraftment of dominant bacteria, such as *Faecalibacterium*, which was not identified without repeated FMT (Wrzosek et al., 2018). The suggested maximum volume for the administration of FMT is 2% of body weight, especially for the mouse. The appropriate amount of fecal infusion is challenging to define. Agreement varies such as 100 µl per dose, (Sivan et al., 2015) 200 µl per dose (Sun et al., 2019) 300 µl per dose (Ericsson et al., 2017) and 400 µl per dose (Lin et al., 2019). FMT performed *via* gastric gavage is advised to infuse feces on three consecutive days, beginning immediately after discontinuation of antibiotics and placement on untreated drinking water (Ericsson et al., 2017) corrected disturbed the gut microbiota (Le Bastard et al., 2018). Overall, it is suggested that repeated gavages once a week for 12 weeks can be a good approach to maintain the human donor microbiota population for 12 weeks (Hintze et al., 2014). The human gut microbiota was reported in recipient mice four weeks post-FMT, irrespective of the FMT approach (Wrzosek et al., 2018). Presently, it is not clear how many FMT dosages are essential to sustain the donor microbiota in the recipient in the long-term or if any association exist between FMT dosages and the donor’s sustainability microbiota. Therefore, upcoming work is desired to conclude the optimal dose. It also not fully defined whether a specific microbial community composition affects the efficiency of FMT. Forthcoming studies might also check this parameter to evaluate the efficacy of FMT at numerous time points and dosages.

## 6 Delivery Routes of FMT

### 6.1 Oral-Gastric Gavage

There is no general agreement on the best approach for delivering fecal microbiota or on the optimal volume. However, oral gastric gavage is a common and preferred method of defined oral dosing for FMT experiments in mice. Fecal suspension may be administered straight into the stomach of mice *via* a technique called oral-gastric gavage (Sivan et al., 2015). In this method, a stainless-steel bulb tilted gavage needle or a flexible cannula or tube is attached to a syringe and used to deliver the fecal suspension into the stomach. The suggested needle or vessel dimension should be equivalent to the distance from the mouth to exactly after the last rib of the abdominal. Before oral gastric gavage, any anesthesia is not recommended, as this will surge the threat of aspiration pneumonia in mice (Turner et al., 2011). Simple oral-gastric gavage administration of mouse donor microbiota effectively remodels gut microbiota in mouse recipients (Zhang et al., 2015; Rausch et al., 2016). This technique remains the most extensively used method for FMT. It also allows for a quicker transfer of feces. Obstacles linked with this method include unintentional administration into the trachea, bronchial pneumonia, esophageal injury or trauma, stomach rupture, and weight loss (de Meijer et al., 2010; Arantes-Rodrigues et al., 2012; Kinder et al., 2014). Stress-induced by this method (Balcombe et al., 2004) can enhance morbidity and mortality in mice.

### 6.2 Enema

Per rectum administration of fecal materials by enema is less common in animals than in humans. FMT by enema may be a practical approach to reduce necrotizing enterocolitis progression. Enema reduces intestinal inflammation and increases intestinal barrier function in mice (Liu et al., 2020). For performing enema, a 4% chloral hydrate/kg body weight solution should be injected intraperitoneal to induce mild anesthesia. The mice should be positioned into the horizontal situation, followed by placement of a plastic tube of 2 mm inner diameter. After applying paraffin oil onto the tube surface, the tube inserts into the colon through the anus approximately 4 cm. The fecal suspension solution should be injected slowly (Zhou et al., 2019). FMT administered *via* oral gavage can be nasty in taste and can also cause gastric irritation. Rectum favors a reproducible absorption course. Rectum has little enzymatic actions as compared to other parts of the gut. Further, it is anatomically relatively easy for rectally administered FMT to spread the distal portion of the colon than the proximal part of the colon. The disadvantage of enema is associated with leakage of the rectum, unpredictable absorption, and risk of injury to the rectum’s inner lining that could lead to infection.

### 6.3 Cohousing

One of the most straightforward approaches to evaluating an involved gut microbiome’s effect on a documented phenotype is the cohousing of affected and unaffected animals already harboring complex microbial populations. Cohousing works on the hypothesis that the gut microbiome of affected animals will be transferred to cagemates, probably *via* stochastic contact to gut microbiota-associated microbes in the environment or entire coprophagy (Ivanov et al., 2008). Cohousing can also be achieved using groupings of colonized and GF mice as a means of assessing the ability of microbial communities to colonize the GF mice (Seedorf et al., 2014). As with FMT, GF mice can be co-housed with colonized mice at various ages to determine the effects of early life events in the adult host (Hansen et al., 2012). Cohousing offers numerous logistical benefits to other methods comprising negligible cost and expertise. However, it is not recommended when donor and recipient are fed different diets consist either high-fat high-sucrose or crude extract of camu camu (Anhe et al., 2019). Cohousing also cannot be executed for human microbiota transfer to mice (Llopis et al., 2016). It also depends on passive, partial, and selective exchanges of microbes between co-housed mice. As a result, a lack of phenotype transfer between co-housed mice does not necessarily remove the involvement of the gut microbiota to the phenotype. By contrast, the positive transmission of specific microbiota directly through oral gavage or other techniques to recipient mice offers direct and clear evidence of a microbial influence (Bel et al., 2014).

## 7 Preparation of Recipient for FMT

### 7.1 Age of Recipient

The age of the recipient determines significant engraftment of donor bacteria (Le Roy et al., 2018). Donor microbiota engrafted better in 3-week old mice compared to 8-week old mice. FMT is more competent if the recipient’s microbiota displays lower richness (Ericsson et al., 2017). Gut microbiota has been less diverse at weaning, probably associated with diet transition from mother milk to a solid diet (Schloss et al., 2012). Microbial diversity surges with age (Zhang et al., 2015) and reaches stability around 8-week of age (Laukens et al., 2016). The immune system is not much prone to alteration by the bacterial influx, perhaps post 8 weeks of age, because of improved stability of the immune system and gut microbiome homeostasis (Laukens et al., 2016). Bowel cleansing is reported more effective in weaning mice (3 weeks old) than in adults (8 weeks old) (Le Roy et al., 2018). Further, the antibiotic treatment causes a long-term alteration in the gut microbiome at the weaning than adult stage (Cox et al., 2014). Considering the provided reports, weaning might be a window of opportunity for FMT.

### 7.2 Methods of Depleting Recipient Microbiota

#### 7.2.1 Antibiotics Administration

The gut microbiota acts as a barricade against the invasion of external microorganisms. The basis of the antibiotic treatment is to drop the gut microbiome load in the recipient to ease competition for the repopulating gut microbiome from the donor. The success of FMT is more reliant on the recipient’s microbial load than the composition of the donor’s feces. Low bacterial load in the recipient can help in better colonizing of donor microbiota (Sarrabayrouse et al., 2020). Antibiotics administration decreases the microbial load in the recipient that would favor better engraftment of donor microbiota (Sarrabayrouse et al., 2020). FMT effectiveness in mice could be higher in the antibiotic treated gut than bowel cleansing treated gut and untreated gut (Ji et al., 2017). Antibiotics have a profound effect on the gut microbiota of recipients; therefore, various concoctions of antibiotics, as well as routes of administration, may demonstrate variable efficacy (Johnson et al., 2004). Treatment with broad-spectrum antibiotics is commonly used to deplete the gut microbiome of mice before FMT. Due to variances in the mechanism of action, antibiotics can selectively diminish different microbes. Multiple alternating antibiotic concoctions courses may help allow stable engraftment of the human gut microbiome in recipient mice (Hintze et al., 2014). The specific antibiotic can change the composition of the gut microbiome to recognize bacterial classes related to different phenotypes (Schubert et al., 2015; Zackular et al., 2016). Some antibiotic treatment protocols additionally include antifungals to avoid fungal overgrowth (Grasa et al., 2015; Zakostelska et al., 2016). Investigators have used numerous antibiotic régimes, which differ in the recipe, dosage, and treatment length (**Table 1**). Often, antibiotics are diluted in drinking water, and mice are allowed to drink during treatment; therefore, actually delivered doses can differ. Water solutions are endorsed to make freshly and change once or twice a week (Smith et al., 2013). Many also supplement sweeteners such as sugar or Kool-aid drink mix to disguise any antibiotics’ bitterness (Baldridge et al., 2015; Emal et al., 2017). Still, studies of mice avoided drinking water and becoming dehydrated when provided antibiotics with sweeteners or sugar (Reikvam et al., 2011; Zakostelska et al., 2016). Oral gavage can stop dehydration and permit the delivery of antibiotics. The oral gastric gavage method is occasionally used alone or mixed with delivery in drinking water (Reikvam et al., 2011). Broad-spectrum antibiotics deplete intestinal microbiota. However, leftover antibiotics might persist in recipients and might affect the engraftment of donor bacteria; thus, FMT is often performed one or two days after the last day of antibiotic treatment. Depending upon the starting bacterial diversity, antibiotic concoction, and length of treatment, resistance to antibiotics may confound results in FMT research.

**Table 1:** Antibiotics regimens for depletion of the gut microbiota in recipient mice.

Antibiotics administration	Antibiotics concoction	Dosage	Duration	Add-ons	Depletion of gut microbiota	References
**Drinking water**	Metronidazole + Vancomycin	1.0 + 0.5 g/L	10 weeks			(Atarashi et al., 2011)
	Metronidazole + Ciprofloxacin	1.0 + 1.0 g/L	2 weeks	Grape Kool aid (20 g/L)	A ~10^5^- to 10^6^-fold reduction in fecal bacteria	(Josefsdottir et al., 2017)
	Penicillin + Streptomycin	2000U/mL +2 mg/mL	3 days			(Zhou et al., 2017)
	Ampicillin + Vancomycin + Polymixin	1.0 + 0.5 + 0.1 g/L	4 weeks		Depletion in microbiota noticed	(Kim et al., 2017)
	Ampicillin + Cefoperazone + Clindamycin	1.0 + 1.0 + 1.0 + 1.0 mg/mL	7 days		Significant reduction in bacterial diversity	(Staley et al., 2017)
	Neomycin + Vancomycin + Invanz	1.0 + 1.0 + 1.0 mg/mL	7 days		Significant reduction in bacterial diversity	(Staley et al., 2017)
	Neomycin + Metronidazole + Vancomycin	1.0 + 1.0 + 0.5 g/L	7 days			(Kinnebrew et al., 2010)
	Streptomycin + Colistin + Ampicillin	5 + 1 + 1 g/L	6 weeks	2.5% sucrose		(Sawa et al., 2011)
	Ampicillin + Neomycin + Streptomycin + Vancomycin	1.0 + 1.0 + 1.0 + 0.5 mg/mL	4-5 weeks			(Khosravi et al., 2014)
	Metronidazole + Vancomycin + Cefoxitin + Gentamicin	1.0 + 1.0 + 1.0 + 1.0 mg/mL	10 days		Significant nonappearance of aerobes and 10^5^- to 10^6^-fold drop in anaerobes	(Ganal et al., 2012)
	Ampicillin + Kanamycin + Metronidazole + Vancomycin	1.0 + 1.0 + 1.0 + 0.5 g/L	3 weeks		A 10^3^ drop in gut bacteria	(Gury-BenAri et al., 2016)
	Ampicillin + Neomycin + Metronidazole + Vancomycin	1.0 + 1.0 + 1.0 + 0.5 g/mL	7 days		A 10^5^- fold drop in fecal microbes	(Ochoa-Reparaz et al., 2009)
			2 weeks	Grape Kool aid (20 g/L)	A ~10^5^- to 10^6^-fold reduction in fecal bacteria	(Josefsdottir et al., 2017)
		1.0 + 1.0 + 1.0 + 0.35 g/L	2 weeks	Grape Kool-Aid (25 g/L)		(Emal et al., 2017; Thackray et al., 2018; Burrello et al., 2018)
		1.0 + 1.0 + 1.0+ 0.5 g/L	4 weeks			(Li et al., 2017; Durand et al., 2018)
			1 month	3% sucrose	∼99% of gut bacteria were removed	(Yan et al., 2016)
**Intraperitoneal injection**	Ampicillin + Neomycin + Metronidazole + Vancomycin	1.0 +1.0 + 1.0 + 1.0 g/L (200 µL/day)	7 days		No effect on gut microbes	(Ochoa-Reparaz et al., 2009)
**Intramuscular injection**	Ampicillin + Metronidazole + Vancomycin	1.0 + 1.0 + 0.5 g/L (100 µL/day)	4-6 days		Significant reduction in aerobe and anaerobes	(Wu et al., 2014)
**Oral-gastric gavage**	Ampicillin + Neomycin + Metronidazole + Vancomycin	1.0 + 1.0 + 1.0 + 0.5 g/L (200 µL/day)	7 days		A 10^6^- fold drop in fecal bacteria	(Ochoa-Reparaz et al., 2009)
	Ampicillin + Neomycin + Metronidazole + Vancomycin + Gentamicin	1.0 + 1.0 + 1.0 + 0.5 + 1.0 g/mL (200 µl/day)	3 days		Nearly complete reduction of gut bacteria	(Kelly et al., 2015)
	Bacitracin + Neomycin + Streptomycin	200 mg/antibiotics/kg body weight	3 days		13-fold decrement in SCFA levels	(Fernandez-Santoscoy et al., 2015)
	Ampicillin + Metronidazole + Vancomycin	2.5 + 2.5 + 1.25 mg/L (200 µl/day)	3 days			(Sun et al., 2019)
	Neomycin + Bacitracin	20mg/antibiotics/mouse	7 days	Pimaricin (5 µg/mouse)	1000-fold reduction in gut bacteria	(Grasa et al., 2015)
	Ampicillin by drinking water Neomycin + Metronidazole + Vancomycin by oral-gastric gavage	1.0 g/L 100 + 100 + 50 mg/kg/mouse/12 h	17 days	Amphotericin B (1 g/L)		(Hintze et al., 2014)
**Drinking water+ Oral-gastric gavage**	Ampicillin + Neomycin + Metronidazole + Vancomycin	10 mg/daily/antibiotics/oral-gastric gavage 1.0 + 1.0 + 1.0 + 0.5 g/L in drinking water	5 days 7-10 days		A million-fold reduction in gut bacteria	(Kuss et al., 2011)
	Kanamycin + Metronidazole + Vancomycin + Gentamicin Colistin +	0.35-4 + 2.15 + 0.45 + 0.35 mg/mL + 8500 U/mL (100 µl daily by oral-gastric gavage) 50-fold dilution for all antibiotics excluding vancomycin (0.5 mg/mL) in drinking water	1 week Till sacrifice		Significant reduction in gut bacterial	(Stefka et al., 2014)
	Metronidazole + Colistin + Streptomycin by oral-gastric gavage and Vancomycin by drinking water	0.4 + 0.3 + 2 mg/mL 0.25 mg/mL	2 weeks	Amphotericin B (20 μg)	Reduction in bacterial diversity but bacterial load remain unchanged	(Zakostelska et al., 2016)
	Streptomycin by oral gavage Ampicillin by drinking water	20 mg/mouse 1 g/L	1-2 weeks		Decrement in bacterial load by ~1000 copy number	(Kim et al., 2018a)
	Streptomycin by oral-gastric gavage Followed by Ampicillin + Neomycin + Vancomycin + Metronidazole in drinking water	100 mg/mouse 1.0 + 1.0 + 1.0 + 0.5 g/L	One dose More than 7 days	1% sucrose		(Kernbauer et al., 2014)

#### 7.2.2 Polyethylene Glycol Administration

Bowel cleansing with laxative based methods such as PEG is an alternative approach for removing gut microbiota before implantation of the donor gut microbiome. The PEG increases the volume of fluids *via* osmotic flow and washes out the luminal bacteria. It also introduces oxygen into the colon, which generally harbors anaerobic bacteria ecosystem and declines the nutrition for them (Strocchi et al., 1990). The higher dose of PEG through oral gastric gavage perhaps permitted a more efficient bowel clean. Recent findings suggested 170 mg of PEG for 4 times at 20 minutes intervals (Wrzosek et al., 2018) and 93 mg of PEG for 5 times at 30 minutes intervals (Le Roy et al., 2018) for clearing the gut and reducing the bacterial load in adult and young mice, respectively. PEG alone can lead to a similar decrease in the quantity of a combination of antibiotics. The PEG method is easy and straightforward to use for diminishing resident bacteria from the gut. The PEG solution causes electrolytes disturbance, dehydration (Yamano et al., 2016; Tropini et al., 2018), and change in the protective mucus lining of the colon, which leads to diarrhea in recipients (Johansson et al., 2014; Jalanka et al., 2015).

In contrast with antibiotics, laxatives temporarily decrease the abundance of gut bacteria ecosystem in humans (Jalanka et al., 2015) and mice (Ji et al., 2017). Bowel cleansing also maintained indigenous microbiota post-treatment compared to antibiotics (Ji et al., 2017).

#### 7.2.3 Germ-Free Mice

Germ-free (GF) animals provide a valuable investigational tool for inspecting connections between a host and its GM. GF animals free from bacteria, viruses, fungi, protozoa, and parasites, throughout their lifespan (Al-Asmakh and Zadjali, 2015). The physiological conditions in the GF gut are different from wild type and specific pathogen-free mice gut. Precisely, increased mucin, pH, urea and O_2_, and low or lack of short-chain fatty acids (SCFAs) (Al-Asmakh and Zadjali, 2015) are among the many typical variances that can have a substantial impact on gut microbiota engraftment (Gillilland et al., 2012; Tomas et al., 2013). Along with stabilization in colonization, GF mice’s innate and adaptive immune system requires 30 days to be functional (El Aidy et al., 2013). GF mice must often be examined for contamination using a combination of bacterial culture, microscopy, serology, gross morphology, and sequencing-based detection techniques (Fontaine et al., 2015; Nicklas et al., 2015).

Additionally, the preservation of GF mice in isolators are quite expensive and limit wide application. The cost and management of isolators may make it impracticable or challenging to conduct behavioral testing or pathological study using GF mice. Overall, these parameters should be considered for planning experimental designs and interpreting results related to microbiota composition analysis and phenotype transmission (Le Roy et al., 2018).

## 8 Engraftment of Gut Microbiota Post-FMT

The engraftment of the gut microbiome can be examined by alpha and beta diversity. The extent of donor engraftment can be tracked using a Bayesian Source Tracker algorithm, an OTU-based approach (Knights et al., 2011; Staley et al., 2016; Staley et al., 2017). Several studies reported success in the donor microbiota colonization post-FMT with a varied range of engraftment (**Table 2**). Transfer of human gut microbiota into GF mice by FMT results in the successful transfer of 85-88% of genera after seven days (Turnbaugh et al., 2009; Staley et al., 2017). Approximately 52-58% of donor mice microbiota successfully transferred in recipient mice post one week of the last dose of FMT and engraftment was maintained for the next three weeks (Ericsson et al., 2017). Further, 57-68% human donor microbial population could be attained post weekly gavage for 12 weeks. Staley and his research group reported engraftment ranging from 63.4% to 87.9% to specific pathogen-free mice at day three post single dose of antibiotics cocktail. ^76^ While engraftment fell marginally post day 3, high levels of donor similarity were retained for the three weeks. Multiple doses of antibiotics cocktail increase the percentage of engraftment compared to a single dose of antibiotics, but it did not increase significantly. The microbiota in control mice without antibiotics cocktail treatment displayed higher similarity (89.4% to 95.0%) to their microbiota post-FMT rather than donor microbiota, suggesting antibiotic treatment is required for an effective FMT (Turnbaugh et al., 2009; Staley et al., 2017).

**Table 2:** FMT dosage details and success rates of FMT.

Host	Recipient	Age of recipient	Time of FMT	Fecal content	FMT method	FMT dose	Duration of FMT	FMT success rate	References
**Human**	Mice	7 weeks	12hr after microbiota depletion	Frozen fecal material reconstituted in sterile saline	Oral-gastric gavage		Weekly for 12 weeks	57-68% after 12 weeks	(Hintze et al., 2014)
**Human**	Mice/GF mice	6-8 weeks	Immediately after microbiota depletion	Frozen fecal material amended with 10% glycerol	Oral-gastric gavage	100 µL	Single dose	~60% after 3 days	(Staley et al., 2017)
**Human**	GF Mice	5-7 weeks	No microbiota depletion before FMT	Freshly voided fecal sample diluted in reduced PBS	Oral-gastric gavage	200 µL	Single dose	88% after 7 days	(Turnbaugh et al., 2009)
**Humanized GF Mice**	GF Mice	10 weeks	No microbiota depletion before FMT	Frozen fecal diluted in reduced PBS	Oral-gastric gavage	200 µL	Single dose	85% after 7 days	(Turnbaugh et al., 2009)
**C57BL/6N Mice**	C57BL/6N Mice	6-10 weeks	24hr after microbiota depletion	Fresh fecal pellets suspended in reduced PBS	Oral-gastric gavage	100 µL (Twice a day for first 2 weeks and later once a week)	Till natural death or sacrifice	2 weeks after first dose of FMT	(Barcena et al., 2019)
**Mice**	Mice	7 weeks	48hr after microbiota depletion	Feces were suspended in PBS	Oral-gastric gavage	200 µL/day	3 days		(Le Bastard et al., 2018)
**C57BL/6J or WSB/EiJ Mice**	C57BL/6J Mice	4 weeks	Immediately after microbiota depletion	Dried fecal material was suspended in PBS	Oral-gastric Gavage	100 µL	Single dose		(Nazmul Huda et al., 2020)
**Balb/c Mice**	C57BL/6Mice	3-5 months	5hr after microbiota depletion	Fecal samples were stored in 10% glycerol at -80°C until use	Oral-gastric Gavage	200 µL	Single dose	19.21%	(Freitag et al., 2019)
**C57BL/6 Mice**	C57BL/6 Mice	6 weeks	Immediately after microbiota depletion treatment	Fresh and frozen fecal samples diluted in sterile water prior FMT	Oral-gastric gavage	300 µL/day	3 days	58-90%	(Ericsson et al., 2017)

Interestingly, not all the bacteria in the donor stool have equal colonization capacity. Bacteriodetes phylum was more successful in colonization compared to Firmicutes phylum (Hintze et al., 2014). It is also important to note that at least some microbiota interactions with the host immune system can only be mediated by host-specific microbiota. The colonization of mice with human microbiota does not fully restore microbiota-associated colonization resistance against some pathogens (Chung et al., 2012; Le Roy et al., 2018).

## 9 Impact of Recipient Immune Status on FMT

The gut microbiota alterations post-FMT employ an intense effect on the immune status of the recipient. It can be a crucial parameter in the success of FMT-based therapeutics. A study has shown that mice without CD4^+^ Foxp3^+^ T-regulatory cells were unable to resolve CDI post-FMT and exacerbated inflammation with failure of engraftment of donor microbiota (Littmann et al., 2021). However, removing B-cells, CD8-positive T cells, Th17, and Th1 cells did not affect the success of FMT in CDI mice (Littmann et al., 2021). Success of FMT might involve other immune cells such as innate lymphoid cells and mucosal-associated invariant T cells that regulate host gut immune homeostasis (Fan et al., 2019; Ioannidis et al., 2020). Host-derived inflammatory byproducts such as reactive oxygen and nitrogen species promote the growth of inflammation-tolerant pathogens (Winter et al., 2013). Further, inflammation-induced metabolites ethanolamine, nitric acid, formic acid, and lactic acid also simultaneously support the growth of inflammation- tolerant bacteria (Thiennimitr et al., 2011; Spees et al., 2013; Hughes and Winter, 2016; Gillis et al., 2018) and may prevent engraftment of microbiota (Littmann et al., 2021). Transplantation studies demonstrated that outcome of grafts is driven by the compatibility of major histocompatibility complex [MHC, called human leukocyte antigen (HLA) in human] within and between species (Trivedi et al., 2007; Ayala Garcia et al., 2012). Interestingly, MHC class II and HLA-DQ2 expression influences gut microbiota composition in mice (Kubinak et al., 2015; Khan et al., 2019) and human (Olivares et al., 2015) respectively. Therefore, it is probable that incompatibilities with human microbiota transplantation into mice are also influenced by MHC when introducing foreign microbes. Though this is not the only contributing factor, it can be an important consideration in FMT studies. Overall, these reports suggested that the recipient’s specific immune status can exploit the effectiveness or success of FMT. A more extensive characterization of host immune status is needed to understand its role in the engraftment of donor microbiota.

## 10 Fecal Components Other Than Bacteria for FMT

Most studies on FMT have focused explicitly on the bacteria component of the microbiota. Even though this is a crucial feature, FMT comprises the transfer of more than bacteria. Viruses, fungi, and microbial metabolites are also components of feces and may affect the recipient (Bojanova and Bordenstein, 2016). Ott et al. demonstrated that fecal suspension of the donor was influential in the treatment of recurrent CDI patients. They reported similarity in phage community six weeks post-FMT in recipient compared to donor (Ott et al., 2017). Gavage of phages of a high-fat diet-fed donor helped in reducing small intestinal bacterial overgrowth in high-fat diet-fed recipient mice (Lin et al., 2019). Patients with recurrent CDI displayed fungal dysbiosis. Improvement after FMT was related to a higher abundance of donor-derived *Saccharomyces* and *Aspergillus* in recipients (Zuo et al., 2018). Microbial metabolites such as short-chain fatty acids (SCFAs) have been shown to noticeable anti-inflammatory and T cell–inducing functions in the host (Arpaia et al., 2013; Smith et al., 2013). Further, FMT rich in SCFAs supplemented with butyric acid effectively managed ischemic stroke (Chen et al., 2019). Understanding link between donor non-bacterial components and host-microbiota throughout the intestine is a crucial next footstep in discovering the prospective of FMT based treatments for numerous forms of gut dysbiosis.

## 11 Conclusions

Despite the many studies using FMT to test the causal link of the microbiome in diseases, a multitude of variables of FMT procedures differ between research labs and institutions, and there is no scientific harmony on the standard methodology. An evidence-based approach and routine assessment of stool and FMT preparation protocol are required to maximize FMT effects and ensure the reproducible outcome.

## Author Contributions

YZ and SB conceived the study, SB wrote the manuscript. YZ, YD, and HP assisted in writing the manuscript. All authors contributed to the article and approved the submitted version.

## Funding

This work was supported by the NINDS through R01 NS102633-04.

## Conflict of Interest

The authors declare that the research was conducted in the absence of any commercial or financial relationships that could be construed as a potential conflict of interest.

## Publisher’s Note

All claims expressed in this article are solely those of the authors and do not necessarily represent those of their affiliated organizations, or those of the publisher, the editors and the reviewers. Any product that may be evaluated in this article, or claim that may be made by its manufacturer, is not guaranteed or endorsed by the publisher.
